# Indocyanine green (ICG) fluorescence in robotic hepatobiliary surgery: A systematic review

**DOI:** 10.1002/rcs.2485

**Published:** 2022-12-02

**Authors:** Archit V. Potharazu, Antonio Gangemi

**Affiliations:** ^1^ College of Medicine University of Illinois at Chicago Chicago IL USA; ^2^ Department of Medical and Surgical Sciences Alma Mater Studiorum University of Bologna, Policlinico Sant’Orsola IRCCS Bologna Italy

**Keywords:** abdominal, cholecystectomy, digestive system, fluorescence, gall bladder, general surgery, hepatectomy, hepatobiliary, imaged guided surgery, intraoperative imaging, liver, minimal invasive surgery, tumour

## Abstract

**Background:**

Indocyanine green fluorescence (ICG‐F) stains hepatic tumours and delineates vascular and biliary structures in real‐time. We detail the efficacy of ICG‐F in robotic hepatobiliary surgery.

**Methods:**

PubMed, EMBASE, Web of Science, and Cochrane Central were searched for original articles and meta‐analyses detailing the outcomes of ICG‐F in robotic hepatobiliary surgery.

**Results:**

214 abstracts were reviewed; 16 studies are presented. One single‐institution study reported ICG‐F in robotic right hepatectomy reduced postoperative bile leakage (0% vs. 12%, *p* = 0.023), R1 resection (0% vs. 16%, *p* = 0.019), and readmission (*p* = 0.023) without prolonging operative time (288 vs. 272 min, *p* = 0.778). Improved visualisation aided in attainment of R0 resection in partial hepatectomies and radical gallbladder adenocarcinoma resections. Fewer ICG‐F‐aided robotic cholecystectomies were converted to open procedure compared to laparoscopic cholecystectomies (2.1% vs. 8.9%, *p* = 0.03; 0.15% vs. 2.6%, *p* < 0.001).

**Conclusions:**

ICG‐F improves clinical outcomes in robotic hepatobiliary surgery without prolonging operative time. There is an opportunity to standardise ICG administration protocols, especially for hepatectomies.

## 1 INTRODUCTION

Proper understanding and identification of the anatomy is essential in performing safe hepatobiliary surgery.^1^, ^2^ Enhanced anatomic visualisation can be provided with indocyanine green (ICG) fluorescent imaging and has as such become adopted by a number of surgical specialties.^3^, ^4^, ^5^, ^6^ ICG is a water‐soluble fluorophore that binds to plasma when injected intravenously and fluoresces at 840 nm with a tissue penetration depth of 5–10 mm when excited by near‐infrared light.^7^ Once within circulation, ICG is taken up by hepatic parenchymal cells and subsequently excreted in the bile after 30–45 min.^6^, ^8^ Accordingly, ICG has been used for over 40 years for hepatic function testing by measuring the rate at which the liver clears the injected fluorophore.^9^

In 2009, Ishizawa et al.^10^ reported the use of intravenously injecting ICG to outline the biliary tree in real‐time, thus introducing ICG fluorescent cholangiography. ICG was also noted to be poorly cleared by dysplastic and neoplastic hepatic cells, thus allowing it to stain liver tumours.^11^ Since then, applications of ICG fluorescent imaging have rapidly expanded to include outlining hepatic anatomy, staining liver tumours, assessing bowel perfusion before intestinal anastomosis, assessing for anastomotic leak, and identifying lymphatic draining basins.^3^, ^8^, ^12^, ^13^, ^14^, ^15^ In 2012, Ishizawa et al.^12^ also reported the feasibility of directly injecting ICG into the portal pedicle to positively stain a certain anatomic region of the liver. They reported ICG may also be intravenously injected with a portal vein branch of interest clamped to negatively stain a segment. Given these varied applications, ICG fluorescent imaging has incredible value in hepatobiliary surgery.

The introduction of the Firefly® real‐time near‐infrared imaging system into the Da Vinci Xi platform has further incentivised the usage of ICG fluorescent imaging. As the surgeon can alternate between the white light and near‐infrared channels on the console without looking away from the surgical field, the robotic platform is all the more equipped for biliary imaging, liver tumour identification, anatomical resection, and post hepatic resection identification of biliary leaks.^16^

In this review we aim to detail clinical outcomes associated with the addition of ICG fluorescence imaging to robotic hepatobiliary procedures. The secondary aim of this study is to outline future perspectives on the utility of ICG fluorescence imaging.

## 2 MATERIALS AND METHODS

**FIGURE 1:**
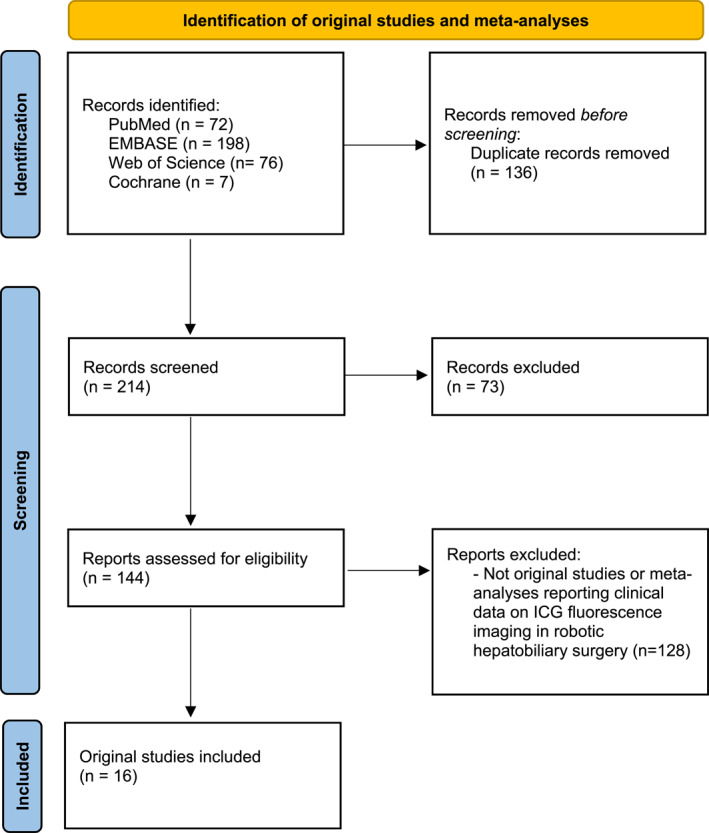
Flowsheet of review methodology per PRIMSA guidelines

A search of the medical literature was made for studies published between 1900 and 15 February 2022 assessing the use of ICG fluorescence imaging in robotic hepatobiliary surgery. PRISMA guidelines for systematic reviews were followed.^17^ Eligibility criteria included articles which (1) were written in English with the full text available; (2) included patients who had undergone a robotic hepatobiliary procedure utilising ICG fluorescence imaging; and (3) provided outcomes regarding the safety, technical feasibility, clinical outcomes, or application of ICG fluorescence imaging in robotic hepatobiliary surgery. Relevant studies were identified via a search of PubMed, EMBASE, Web of Science, and Cochrane Central.^18^ The following search terms were used: (ICG OR indocyanine green) AND (hepatobiliary OR hepatic OR liver OR hepatectomy OR gallbladder OR cholecystectomy) AND (robot OR robotic). After duplicates were removed, all abstracts were screened for full‐text review by two independent reviewers. Following the initial screening stage, identified manuscripts were read to identify original studies and meta‐analyses satisfying the above criteria. Individual case reports, podium presentations, and posters were not included (Figure 1).

Each study was assessed for risk of bias using the Cochrane ROBINS I tool for non‐randomized studies. Assessment was based on risk of bias in confounding, participant selection, intervention classification, deviations in intended interventions, missing data, incomplete measurement of outcomes, and reporting bias. The risk of overall bias was then rated categorically as very low, low, moderate, or high as per the Cochrane guidelines.^18^

## 3 RESULTS

Among the 219 abstracts reviewed, 16 original studies were included (Table 1). Seven studies reported data regarding efficacy of ICG fluorescence staining of liver tumours and anatomical regions for robotic partial hepatectomy; 8 studies reported data regarding rates of biliary anatomy visualisation, conversion to open procedure, and bile duct injury with ICG‐aided robotic cholecystectomy; and 1 study reported a case series of R0 robotic radical gallbladder adenocarcinoma resections with the aid of ICG cholangiography. No study was excluded due to high risk of bias (Table 2). The most common source of potential bias was due to the potential of confounding in the non‐randomized setting. ICG was safely administered in all cases in all studies.

**TABLE 1:** Identified original studies and meta‐analyses

Reference	Design	Procedure (number of cases)	ICG utility^a^	Key outcomes
Hepatectomy				
Marino et al., 2019^22^	Case‐matched comparison	ICG‐aided robotic right hepatectomy (25) Non‐ICG‐aided robotic right hepatectomy (25)	Preoperative administration for tumour staining Intraoperative administration for anatomical delineation ‐ Positive staining (*n* = 8) ‐ Negative staining (*n* = 13) ‐ Counterstaining (*n* = 4) Intraoperative identification of biliary leakages after liver surface irrigation.	ICG‐F reduced the rate of postoperative bile leakage (0% vs. 12%, *p* = 0.023), R1 resection (0% vs. 16%, *p* = 0.019), and readmission (*p* = 0.023) without prolonging operative time (288 vs. 272 min, *p* = 0.778). Three biliary leakages (12%) identified at the liver resection surface in the ICG‐F group following post‐resection irrigation.
Mehdorn et al., 2021^21^	Case series	ICG‐aided robotic atypical liver resection (16) ICG‐aided robotic minor hemihepatectomy (3) ICG‐aided robotic major hemihepatectomy (1)	Preoperative administration for tumour staining	Good intraoperative ICG tumour staining correlated with R0 resection rate (85%). Persistent ICG‐F led to extension after initial resection in one case, thereby achieving an R0 situation.
Franz et al., 2021^19^	Case series	ICG‐aided robotic partial hepatectomy (11) ICG‐aided laparoscopic partial hepatectomy (7)	Preoperative administration for tumour staining	100% detection rate of known lesions. ICG‐F of previously nondetectable lesions affected therapeutic strategy in 27.8% of cases. 39% false‐positive rate
Takahashi et al., 2016^11^	Case series	ICG‐aided robotic partial hepatectomy (2) ICG‐aided laparoscopic microwave ablation (5) ICG‐aided laparoscopy and biopsy (3) Open partial hepatectomy (5)	Preoperative administration for tumour staining	ICG detected all 34/62 superficial lesions but failed to detect any of the 28 deep lesions.
Marino et al., 2020^20^	Retrospective cohort	ICG‐aided robotic partial hepatectomy (40)	Preoperative administration for tumour staining Intraoperative administration for anatomical delineation ‐ Positive staining (*n* = 20) ‐ Negative staining (*n* = 20)	R0 resection rate of 100%. 52 of 59 lesions were identified by ICG. ICG‐F identified more tumours than intraoperative ultrasound and white light alone (52 vs. 43). ICG failed to identify 7 lesions; missed tumours were located deeper than ICG‐F identified tumours (10 mm [range, 6–42 mm] vs. 1 mm [range 1–8 mm]).
Chiow et al., 2021^24^	Case series	ICG‐aided robotic partial hepatectomy (52)	Intraoperative administration for anatomical delineation ‐ positive staining (*n* = 12) ‐ Negative staining (*n* = 40)	When well visualised, the ICG demarcation line was as clear as the ischaemic line in 14/43 cases and clearer in 29/43 cases.
Achterberg et al., 2020^23^	Retrospective cohort	R0 minimally invasive hepatic CRLM resection (8) R1 minimally invasive hepatic CRLM resection (8)	Preoperative administration for tumour staining	8/8 lesions with a positive ICG‐F margin had a positive tumour margin confirmed by histopathology. 7/8 lesions with a negative ICG‐F margin had a negative tumour margin.
Cholecystectomy				
Gangemi et al., 2017^31^	Retrospective cohort, multi‐centre	ICG‐aided RC (676) LC (16 965)	2.5 mg, 45 min prior to surgery	ICG‐aided RC has a lower rate of conversion than LC
Sharma et al., 2017^32^	Retrospective cohort	ICG‐aided RC (96) LC (191)	‐‐	Statistically significantly fewer open conversions were found in the robotic than the laparoscopic group (*p* = 0.03), but odds ratio calculation with multiple logistic regression was non‐contributory (*p* = 0.22)
Daskalaki et al., 2014^27^	Case series	ICG‐aided RC (184)	2.5 mg, 45 min prior to surgery	No conversions occurred, no biliary injuries occurred, and at least 1 biliary structure was identified in 99% of cases
Diana et al., 2017^29^	Prospective cohort	RC (58) with AR navigation (43), ICG‐aid (54), or IOC (52)	0.1–0.4 mg/kg, 45–60 min prior to surgery	The CD‐CBD junction was visualised by ICG in 98.15% of cases; images with ICG were obtained more quickly than with AR navigation or IOC, but were of lower image quality
Spinoglio et al., 2013^26^	Case series	ICG‐aided single‐site RC (45)	2.5 mg, 30–45 min prior to surgery	No conversions occurred, no biliary injuries occurred, and at least 1 biliary structure was identified in all cases
Buchs et al., 2013^30^	Prospective cohort	ICG‐aided RC (23) Non‐ICG‐aided RC (21)	2.5 mg, 30–45 min prior to surgery	Overall operative time with ICG‐aid was shorter for patients with a BMI ≤25, but without reaching statistical significance (*p* = 0.06)
Maker & Kunda, 2017^28^	Case series	ICG‐aided multiport RC (35)	2.5 mg injected at time of intubation	No conversions occurred, no biliary injuries occurred, and critical view was obtained in all patients without use of IOC
Buchs et al., 2012^25^	Case series	ICG‐aided single‐site RC (12)	2.5 mg, 30–45 min prior to surgery	One case required port addition; no perioperative complications occurred; at least one biliary structure was identified in all cases
Radical Resection for Gallbladder Cancer				
Ahmad, 2020^34^	Case series	Robotic radical gallbladder adenocarcinoma resection, entailing central hepatectomy (segments IV‐B and V) and regional lymph node dissection (10)	5 mg, 30–60 min prior to surgery	High ligation of the cystic duct close to the CD‐CBD junction was performed with ICG‐aid and negative margins were achieved in all patients

Abbreviations: CD‐CBD junction, cystic duct‐common bile duct junction; CRLM, colorectal liver metastases; ICG, indocyanine green; ICG‐F, indocyanine green fluorescence; LC, laparoscopic cholecystectomy; RC, robotic cholecystectomy.

^a^ Due to protocol heterogeneity, see Table 2 for specific ICG administration protocols for hepatectomies.

**TABLE 2:** Risk of bias assessment via ROBINS‐I tool^18^

Reference	Confounding	Participant selection	Classification of interventions	Deviations in interventions	Missing data	Measurement of outcomes	Biased reporting	Overall bias rating
Marino et al., 2019^22^	Moderate	Moderate	Low	Low	Low	Low	Low	Moderate
Mehdorn et al., 2021^21^	Low	Low	Low	Low	Low	Low	Low	Low
Franz et al., 2021^19^	Moderate	Moderate	Low	Low	Low	Low	Low	Moderate
Takahashi et al., 2016^11^	Low	Low	Low	Low	Low	Low	Low	Low
Marino et al., 2020^20^	Moderate	Moderate	Low	Low	Low	Low	Low	Moderate
Chiow et al., 2021^24^	Low	Low	Low	Low	Low	Low	Low	Moderate
Achterberg et al., 2020^23^	Moderate	Moderate	Low	Low	Low	Low	Low	Moderate
Gangemi et al., 2017^31^	Moderate	Moderate	Low	Low	Low	Low	Low	Moderate
Sharma et al., 2017^32^	Moderate	Moderate	Low	Low	Low	Low	Low	Moderate
Daskalaki et al., 2014^27^	Low	Low	Low	Low	Low	Low	Low	Low
Diana et al., 2017^29^	Moderate	Low	Low	Low	Low	Low	Low	Moderate
Spinoglio et al., 2013^26^	Low	Low	Low	Low	Low	Low	Low	Low
Buchs et al., 2013^30^	Moderate	Moderate	Low	Low	Low	Low	Low	Moderate
Maker & Kunda, 2017^28^	Low	Low	Low	Low	Low	Low	Low	Low
Buchs et al., 2012^25^	Low	Low	Low	Low	Low	Low	Low	Low
Ahmad, 2020^34^	Low	Low	Low	Low	Low	Low	Low	Low

Takahashi et al.^11^ reported a case series in which ICG fluorescence imaging was used to detect liver tumours in 15 patients. 7.5 mg (concentration 2.5 mg/ml) of solution were administered 0–2 days prior to surgery, with the expectation that the normal hepatic parenchyma would clear the ICG while the tumour cells would take up ICG but fail to excrete it into the bile. The authors recommended administration 24 h prior to surgery, as they found a longer time period resulted in minimal tumour‐tissue contrast whereas a shorter time period resulted in greater false positive signal. All malignant lesions stained hyperfluorescent regardless of pathology. Only two cases were robotic resections while the majority were open or laparoscopic. Interestingly, all 34 lesions in the series defined as superficial were identified by ICG, including 11 not identified by preoperative imaging. However, no lesions defined as deep were identified. This led Takahashi et al. to recommend ICG fluorescence imaging as a key adjunct to intraoperative ultrasound, as the former is specially equipped for the detection of superficial lesions, while the latter can detect deeper lesions beyond ICG's penetration range of 6‐8 mm.^11^

Subsequent case series by Franz et al.,^19^ Marino et al.,^20^ and Mehdorn et al.^21^ reported improved known lesion detection rates of 100%, 88%, and 85%, respectively. Different groups reported different ICG administration protocols for robotic hepatectomy (Table 3). Franz et al. administered ICG 2–10 days, Marino et al. 5 days, and Mehdorn et al. 1 day prior to surgery. All groups used a weight‐based dosing regimen of 0.5 mg/kg compared to Takahashi et al.'s significantly smaller 7.5 mg dose. All malignant tumours did not stain homogenously: well differentiated hepatocellular carcinomas (HCCs) exhibited a total‐stain pattern, whereas colorectal liver metastases (CRLMs) exhibited a rim‐stain pattern, with fluorescent uptake surrounding, but not within, the lesion. Moderately‐ and poorly‐differentiated HCCs exhibited staining patterns ranging from rim‐type^22^ to partial‐type^20^, ^22^ to total‐type.^21^ Marino et al. also noted missed tumours were located at a mean depth of 10 mm (range 6–42 mm) versus 1 mm (range 1–8 mm) for identified tumours. False‐positives were reported in older patients and patients with cirrhotic or fibrotic livers, likely due to impaired ICG clearance by the hepatic parenchymal cells. Franz et al. suggested cirrhotic livers require 7–10 days to clear ICG.

**TABLE 3:** Indocyanine green administration protocols for hepatectomies and associated imaging quality

Reference	Preoperative protocol	Tumour staining image quality	Intraoperative protocol	Anatomic staining image quality
Marino et al., 2019^22^	0.5 mg/kg body weight, 5 days prior to surgery	Total fluorescence in HCC (*n* = 8) Rim fluorescence in CRLM (*n* = 18) Partial fluorescence (*n* = 6) and rim fluorescence (*n* = 5) in poorly‐ or moderately‐differentiated HCC	2.5 mg (0.25 mg/ml) for positive staining (*n* = 8) and counterstaining (*n* = 4) 2.5 mg (2.5 mg/ml) for negative staining (*n* = 13) when tumour‐bearing portal vein occluded by tumour invasion or preoperative portal embolisation.	Hepatic boundary visualisation was faster with positive staining versus negative staining or counterstaining (110 vs. 390 s and 220 s). Negative staining offered better visualisation in patients with previous liver resection or chemotherapy‐induced steatohepatitis.
Mehdorn et al., 2021^21^	25 mg (0.5 mg/ml), 1 day prior to surgery	Total fluorescence in HCC, regardless of grading (*n* = 5) Rim fluorescence in non‐HCC metastases (*n* = 7) Poor accumulation, whole‐liver staining, or ubiquitous accumulation in cirrhotic liver (*n* = 7)	‐‐	‐‐
Franz et al., 2021^19^	0.5 mg/kg body weight, 2–10 days prior to surgery (mean 4 days)	18/18 tumours stained (sensitivity 100%) ‐ HCC (*n* = 9), CRLM (*n* = 4) False‐positive signal of 39% (specificity 72%) ‐ Identified in cirrhotic tissue and regenerative liver parenchyma	‐‐	‐‐
Takahashi et al., 2016^11^	7.5 mg, 0–2 days prior to surgery	All malignant superficial lesions were hyperfluorescent on ICG‐F Of benign lesions, focal nodular hyperplasia was hyperfluorescent, while haemangioma was hypofluorescent. Best contrast was obtained with administration 24 h prior to surgery.	‐‐	‐‐
Marino et al., 2020^20^	0.5 mg/kg body weight, 5 days prior to surgery	Total fluorescence in well‐differentiated HCC (*n* = 12) Rim fluorescence in CRLM (*n* = 27) Partial fluorescence in poorly‐ and moderately‐differentiated HCC and other tumours (*n* = 20)	2.5 mg (0.25 mg/ml) for positive staining (*n* = 20) 2.5 mg (2.5 mg/ml) for negative staining (*n* = 20) when tumour‐bearing portal vein occluded by tumour invasion or preoperative portal embolisation.	Hepatic boundary visualisation was faster with positive staining versus negative staining (90 vs. 375 s). However, the negative staining method generated higher quality images, was rated to be more comfortable, and allowed selectivity in cirrhotic tumours
Chiow et al., 2021^24^	‐‐	‐‐	5 mg for both positive and negative staining (dilution not reported)	The ICG demarcation line was clearly seen in 6/12 cases with positive staining and 37/40 cases with negative staining. Negative staining was also less technically challenging
Achterberg et al., 2020^23^	10 mg (5 mg/ml), 1 day prior to surgery	Rim fluorescence in CRLM (*n* = 16) ‐ Atypical fluorescence around previously ablated liver tissue (*n* = 1)	‐‐	‐‐

Abbreviations: CRLM, colorectal liver metastases; ICG, indocyanine green; ICG‐F, indocyanine green fluorescence.

Marino et al. and Mehdorn et al. reported R0 resection rates of 100% and 85% and cited the value of ICG in providing real‐time visualisation of the tumour. Achterberg et al.^23^ noted a strong correlation between ICG resection margin and histopathological tumour margin—8/8 lesions in their retrospective analysis with a positive fluorescence margin had a confirmed positive tumour margin and 7/8 lesions with a negative fluorescence margin had a confirmed negative tumour margin.

Marino et al.^22^ also performed a case matched comparison between patients receiving robotic right hepatectomies with and without ICG fluorescence. They found ICG fluorescence reduced the rate of postoperative bile leakage (0% vs. 12%, *p* = 0.023), R1 resection (0% vs. 16%, *p* = 0.019), and readmission (*p* = 0.023) without prolonging operative time (288 vs. 272 min, *p* = 0.778).

ICG is also gaining popularity in hepatic surgery as a tool for anatomic visualisation. Overall, two imaging techniques have been described. Positive staining of a region of interest involves direct intraoperative ICG injection into the portal pedicle, whereas negative staining involves clamping of the pedicle and injection of ICG into the portal vein or intravenously. Marino et al.^20^ noted faster visualisation with positive staining compared to negative staining (90 s from time of injection vs. 375 s) but with reduced image quality. They also noted negative staining was rated as more comfortable by surgeons and allowed selective visualisation even in cirrhotic tumours. Chiow et al.^24^ also reported more positive results with the negative staining technique. Their group initially used the positive staining technique via injection directly into the portal pedicle. However, the procedure was technically challenging and the ICG demarcation line was only successfully visualised in 6/12 cases. As such, they transitioned to the negative staining technique and found significant improvement; the ICG demarcation line was successfully visualised in 37 out of 40 cases.

Buchs et al.^25^ were the first to report data regarding biliary structure visualisation with ICG cholangiography in robotic cholecystectomy in their 2012 case series. The cystic duct (CD), common hepatic duct (CHD), common bile duct (CBD), and cystic duct‐common hepatic duct (CD‐CHD) junction were visualised in 91.7%, 33.3%, 50%, and 25% of cases before dissection of Calot's triangle, respectively. At least one biliary structure was visualised in all cases. Post dissection these structures were visualised in 100%, 66.7%, 83.3%, and 58.3% of cases, respectively.

Replication of Buchs et al.'s work in the following years reported higher rates of visualisation. Spinoglio et al.^26^ reported visualisation of the CD, CHD, CBD, and CD‐CHD junction in 93%, 88%, 91%, and 88% of cases before dissection of Calot's triangle, respectively. Daskalaki et al.^27^ reported similar rates of visualisation of 97.8%, 94%, 96.1%, and 83.6%, respectively. Notably, rates of visualisation were reduced to 91.6%, 79.1%, 79.1%, and 75%, respectively, in patients with acute cholecystitis. This finding is consistent with ICG fluorescence's limited penetration of deep tissue; the dense inflammatory tissue associated with acute cholecystitis likely prevented adequate visualisation in some cases. However, Daskalaki et al. still reported an overall rate of visualisation of at least one biliary structure of 99%. Maker and Kunda^28^ again confirmed this finding their case series in which ICG cholangiography was safely able to aid the surgeon in obtaining the critical view in every case without the need for IOC. In summary, ICG cholangiography routinely delineates all structures of the extrahepatic biliary tree, thereby resulting in improved recognition of patient anatomy.

Diana et al.^29^ studied rates of visualisation with ICG cholangiography alongside classic IOC and a novel intraoperative augmented reality (AR) imaging modality based on three‐dimensional reconstruction of preoperative MRCP images. They reported visualising the CD‐CHD junction with AR in 100% of cases, with ICG cholangiography in 98.15% of cases, and with IOC in 96.15% of cases. Although images with ICG fluorescence were rated to be of lower quality than those with AR or IOC, the mean time to obtain images with ICG fluorescence was shorter. Accordingly, ICG cholangiography may serve as an augmentation and not necessarily a replacement for IOC in particularly difficult cases.

Buchs et al.^30^ directly compared ICG‐aided robotic single‐site cholecystectomy to robotic single‐site cholecystectomy without ICG cholangiography. Perioperative data demonstrated no difference in operative time, docking time, rate of conversion, rate of intraoperative complication, blood loss, rate of postoperative complication, or hospital stay. However, a subgroup analysis stratifying patients by BMI suggested a shorter operative time but did not demonstrate statistical significance. In patients with a BMI ≤25 kg/m^2^, operative time for the ICG group was 70 **+/−** 13.1 min and 93.7 **+/−** 32.5 min for the standard group (*p* = 0.06). This prospective cohort study had a total of 44 patients, raising the possibility that the study's power may not have been sufficient to capture the effect size. In patients with reduced to normal adipose tissue, thereby allowing full penetration of ICG fluorescence, ICG cholangiography may potentially reduce OR time given real‐time visualisation of biliary anatomy.

Gangemi et al.^31^ were the first to demonstrate ICG‐aided robotic cholecystectomies had a lower rate of conversion to open procedure compared to standard laparoscopic cholecystectomy. A multi‐centre retrospective cohort comparison between ICG‐aided robotic cholecystectomy at a single centre, laparoscopic cholecystectomy at the same single centre, and laparoscopic cholecystectomy across a large hospital system demonstrated rates of conversion to open procedure of 0.15%, 4.5%, and 2.6%, respectively. Sharma et al.^32^ also found a reduced rate of open conversions in ICG‐aided robotic cholecystectomy compared to laparoscopic cholecystectomy in their single‐centre study. However, they were unable to demonstrate a statistically significant odds ratio using multiple logistic regression.

Gangemi et al.^31^ reported a reduced rate of minor biliary injuries with ICG‐aided robotic cholecystectomy compared to laparoscopic cholecystectomies within a single institution, but this finding was not replicated when compared to laparoscopic cholecystectomies across a large hospital system. Dip et al.'s^33^ meta‐analysis was also unable to demonstrate a statistically significant reduction in bile duct injuries when ICG cholangiography is utilised. Only 2 biliary injuries were identified across 860 ICG‐aided robotic cholecystectomies and 1242 non‐ICG‐aided robotic cholecystectomies. Given the low rate of biliary injury, these studies may have been too underpowered to demonstrate an effect size, should one exist.

Ahmad^34^ published a case series examining the safety of the use of ICG cholangiography in robotic radical resection of gallbladder adenocarcinoma including central hepatectomy with regional lymphadenectomy. ICG was safely administered to all patients and ICG cholangiography was subjectively reported to aid in attainment of a negative cystic duct margin via identification of the CD‐CBD junction as well as in lymphatic clearance around the biliary tree. Ahmad cited the nature of GBC to potentially compromise typical anatomic planes due either to tumour progression or revisitation of an operative field following cholecystectomy. He emphasised the utility of ICG cholangiography in delineating biliary anatomy, thus allowing for identification and resection of key structures despite the presence of inflammatory or carcinogenic adhesions. He also noted the value of ICG cholangiography in preventing bile duct injury despite the aforementioned anatomic difficulties.

## 4 DISCUSSION

The robotic platform is well‐known to carry many advantages with it in modern minimally invasive surgery, including stable three‐dimensional imaging, improved ergonomics, and enhanced dexterity with seven degrees of freedom.^14^, ^35^ A further benefit of the robotic system, as detailed in this review, is the integrated near‐infrared fluorescent Firefly® camera, which allows for real‐time anatomic visualisation with ICG tumour staining, angiography, and cholangiography.

Preoperative administration of ICG, usually in a weight‐based dose of 0.5 mg/kg body weight, multiple days (reported range of 0–10 days) before surgery allows for staining of hepatic tumours. Once in systemic circulation, ICG binds to plasma proteins, including albumin and alpha‐1/beta‐lipoproteins, and is taken up by hepatocytes via organic anions‐transporting polypeptides (OATP) and sodium taurocholate co‐transporting polypeptides (NTCP).^19^ Normal hepatic parenchyma then excretes ICG into the bile. Interestingly, ICG stains hepatic tumours differentially in a pathology‐dependent fashion. Well‐differentiated HCCs, which have a higher expression of OATP and NTCP but lower biliary excretion functionality, stain in a homogenously fluorescent, also referred to as total‐fluorescent, pattern. Moderately‐ and poorly‐differentiated HCCs stain in various fashions.^20^, ^21^, ^22^ CRLMs do not take up ICG, but due to the reduced biliary excretions capabilities of surrounding immature hepatocytes, are stained in a rim‐pattern.^19^ The heterogeneity in ICG administration protocols suggests an opportunity for studies providing clear guidelines. Such guidelines should likely have different recommendations for healthy versus cirrhotic or fibrotic livers, given that the later take more time to excrete, perhaps begetting a need for preoperative administration beyond 7 days prior to surgery.^19^

In robotic liver surgery, identification of ICG‐stained hepatic lesions aids in the attainment of R0 resection. On the superficial surface of the liver, to a depth of 6–8 mm, ICG fluorescence has a high tumour identification sensitivity. The false‐positive rate is dependent upon ICG administration timing, with administration closer to the date of surgery correlated to a higher false‐positive rate due to staining of normal liver parenchyma.^19^ Nevertheless, ICG is a vital adjunct to intraoperative ultrasound given multiple studies report its ability to identify lesions not detected by preoperative imaging or intraoperative ultrasound^11^, ^20^, ^21^, ^22^, ^23^

The versatile imaging modality can also delineate anatomic segments of the liver with negative or positive staining. Positive staining, which involves direct injection of ICG into the tumour‐bearing portal vein, is more technically difficult but provides an image more quickly. Negative staining, which involves systemic injection of ICG with the tumour‐bearing inflow clamped, provides more clear images, especially in cirrhotic or fibrotic livers, but cannot be redone if improperly done the first time, as the entire liver will be stained. Different centres have different preferences for when to use each technique. However, both techniques may provide clearer demarcation lines than the ischaemic line, which is particularly useful in healthy donor patients and patients with liver cirrhosis or fibrosis.^20^, ^24^, ^36^ The key advantage of the robotic platform, its seven degrees of freedom and endowrist capabilities, improve the feasibility of positive staining over the laparoscopic platform. Accordingly, a single centre study found the use of ICG fluorescence imaging in robotic right hepatectomy reduces the rates of post‐operative bile leakage, R1 resection, and readmission without prolonging operative time. These studies should be replicated and expanded in other centres.

ICG cholangiography also provides clear benefits to robotic cholecystectomies. The imaging modality effectively identifies all structures in the extrahepatic biliary tree, thereby allowing surgeons to obtain the necessary critical view of safety for cholecystectomy. This is particularly important in radical resections for gallbladder adenocarcinoma, which include central hepatectomy (segments IV‐B and V) and regional lymphadenectomy. As such procedures are often conducted following incidental diagnosis after cholecystectomy, tissue planes with dense adhesions must be revisited. The clear delineation of anatomy by ICG cholangiography is especially helpful. In addition, various case reports suggest that ICG cholangiography helps identify aberrant biliary anatomy, providing further guidance in unexpectedly difficult procedures.^37^, ^38^, ^39^, ^40^, ^41^, ^42^ The enhanced anatomic visualisation translates to improved clinical outcomes; across multiple studies, ICG cholangiography provided an associated reduction in rates of conversion to open procedure compared both to laparoscopic cholecystectomies and robotic cholecystectomies performed without ICG imaging. These findings coincide with Dip et al.'s^33^ recently published a meta‐analysis, which demonstrated that the addition of ICG cholangiography to robotic cholecystectomy markedly reduces the risk of conversion to open procedure.

Although data regarding bile duct injury is mixed,^43^ future studies would be remiss in not stratifying procedures by use of ICG cholangiography when possible. Pooling these data when subgroup analysis is available would risk eliminating the effect size produced by the improved extrahepatic biliary tree visualisation provided by routine fluorescence imaging. In addition, as with any novel procedure, surgeon skill and comfort will continue to improve with usage, potentially improving already compelling outcomes. Some authors have already advocated for routine use of ICG in robotic hepatobiliary surgery.^44^

Reduction of these negative outcomes in turn may bring a reduction in overall costs. A cost analysis of ICG integration in laparoscopic cholecystectomies suggested the reduced rate of conversion to open procedure, shortened operative time, and reduced rate of bile duct injury helps decrease the cost of the procedure.^45^ The da Vinci Firefly® system as an integrated near‐infrared camera. Therefore, in robotic surgery, the only per‐procedure cost of ICG fluorescence in the cost of the dye itself. It is to be noted that the robotic platform has a high fixed costs and is associated with longer operating time, and therefore higher costs, than the laparoscopic platform. The cost‐benefit analysis between laparoscopic and robotic surgery should be revisited as new techniques, such as the Firefly® continue to capitalize on the platform's potential. Nevertheless, given the improvement in clinical outcomes provided in robotic hepatobiliary surgery, integration of the imaging technique may also reduce overall costs.

Importantly, ICG provides this value while being safe. Compared to the classic intraoperative cholangiogram (IOC)—in which a radiotracer is injected directly into the cystic duct and X‐ray imaging is used to visualise the entire biliary tree—ICG provides real‐time imaging, provides no risk of radiation exposure, and comes with no risk of bile duct injury from cystic duct cannulation.^46^, ^47^, ^48^ The main risk of ICG is that of anaphylaxis, which has a reported rate of 1/80 000.^49^ This risk can be further minimised by preoperative ICG skin testing, exclusion of patients with iodine allergy, and exclusion of patients with hyperthyroidism.

Future use of and indications for ICG fluorescence imaging will no doubt continue to evolve. Intravenous ICG injection could potentially be used to assess newly‐made hepaticojejunostomies for bile leakage, allowing for prompt revisions of anastomotic leakages.^50^ Parallelling the rise of novel targeted cancer immunotherapies, next generation dyes may be conjugated to tumour‐specific antibodies. This modality promises to provide increased selectivity and allow for dual‐channel imaging when used in conjugation with classic ICG.^23^ The continued development and integration of image‐guided robotic surgery may eventually lead to routine integration in the future.^6^ Negative outcomes in a procedure that failed to use ICG when indicated may carry medico‐legal implications should the use of ICG fluorescence imaging become standard of care.

The ability of ICG to outline key anatomic structures may also yield incredible benefits for students. The robotic platform already lends itself well for virtual‐reality based anatomical curricula^51^ and has already been piloted for teaching students urologic anatomy through simulated robot‐assisted radical prostatectomy.^52^ The modern explosion of biomedical knowledge has led to a concurrent reduction in undergraduate anatomy education, prompting a need for innovation in this field.^53^ Early exposure of students to surgery and anatomy through image‐guided surgery on the robotic platform with fluorescence imaging may help spark lifelong interest at a young age.

The major limitation of ICG is the reduced tissue penetration depth.^11^ Patients with an iodine allergy, severe renal function impairment, and hyperthyroidism should not receive ICG.^19^, ^21^ Limitations of this review include the bias of published articles to only report positive results. ICG protocols were inconsistent, particularly with regards to liver imaging, where no consensus protocol exists.^54^ In addition, our review only focussed on ICG fluorescence in robotic hepatobiliary surgery and did not discuss much the impact of fluorescence imaging in open or laparoscopic surgery.

Reasons for the concision of this review are two‐fold. First, we only focussed on the specialty of hepatobiliary surgery. This is a high morbidity field in which innovation may provide significant improvements. Most importantly, constraining the scope improves the clarity of any results obtained. By focussing on the outcomes only in robotic hepatobiliary surgery, we are able to confidently advocate for ICG fluorescence imaging's routine usage. The imaging modality improves clinical outcomes in a high morbidity surgical specialty.

Second, ICG fluorescence imaging is a novel technology, having largely been expanded only in the last 10 years. However, given the relative ease of incorporation in robotic surgery and myriad surgical specialties using ICG imaging, we have no doubt that the literature on this topic will expand. Future reviews may delve more into the indications of ICG imaging. At this point in the timeline of ICG development, we are still in the era of advocacy, not yet widely accepted routine indications.

Further investigation in this field should be geared towards larger prospective or randomized studies comparing hepatobiliary procedures with and without the utilization of ICG fluorescent imaging. Additionally, work is needed, especially for hepatectomies, to develop consistent recommendations for ICG administration protocols. It is plausible that differential recommendations will be needed for patients with and without liver and cirrhosis and fibrosis.^19^ As uptake of ICG imaging increases, head‐to‐head comparisons between open, laparoscopic, and robotic procedures should be revisited.

## CONFLICT OF INTEREST

The authors have no financial conflicts of interest to disclose.

## Data Availability

Data sharing is not applicable to this article as no new data were created or analyzed in this study.
